# Contextualizing Autophagy during Gametogenesis and Preimplantation Embryonic Development

**DOI:** 10.3390/ijms22126313

**Published:** 2021-06-12

**Authors:** Marcelo T. Moura, Laís B. Latorraca, Fabíola F. Paula-Lopes

**Affiliations:** Department of Biological Sciences, Campus Diadema, Federal University of Sao Paulo—UNIFESP, Diadema 09972-270, SP, Brazil; lais_latorraca@hotmail.com

**Keywords:** ART, autophagic, cloning, embryogenesis, oogenesis, reproduction, reprogramming, spermatozoa

## Abstract

Mammals face environmental stressors throughout their lifespan, which may jeopardize cellular homeostasis. Hence, these organisms have acquired mechanisms to cope with stressors by sensing, repairing the damage, and reallocating resources to increase the odds of long-term survival. Autophagy is a pro-survival lysosome-mediated cytoplasm degradation pathway for organelle and macromolecule recycling. Furthermore, autophagy efflux increases, and this pathway becomes idiosyncratic depending upon developmental and environmental contexts. Mammalian germ cells and preimplantation embryos are attractive models for dissecting autophagy due to their metastable phenotypes during differentiation and exposure to varying environmental cues. The aim of this review is to explore autophagy during mammalian gametogenesis, fertilization and preimplantation embryonic development by contemplating its physiological role during development, under key stressors, and within the scope of assisted reproduction technologies.

## 1. Introduction

Mammalian development begins with a plethora of events: fertilization, pro-nuclei formation and syngamy, and the first mitotic division [1,2]. Thereafter, embryos experience multiple rounds of cell divisions during the pre-compactation period under complete maternal developmental control [3,4]. Oocytes provide most cellular components for upcoming blastomeres, which were stockpiled during the oocyte growth [5,6,7]. The embryonic genome activation (EGA) occurs in a species-specific manner [7,8], from the two-cell stage in the mouse up to the eight-to-the-sixteen cell stage transition in sheep and cattle [9]. The EGA landmarks the transition from maternal-to-embryonic developmental control, thus making embryonic cells progressively responsible for providing proteins and organelles [7].

The developing embryo subsequently initiates the cavitation period evidenced by the blastocele formation [4,9]. At the blastocyst stage, the embryo encompasses two morphologically and functionally distinct cell types: the trophectoderm (TE) and the inner cell mass (ICM) [8,9]. The placenta will be derived from the TE lineage. In contrast, the ICM gives rise to the embryo proper, and the germ-line precursors named the primordial germ cells (PGCs). These later cells will migrate to the genital ridges, proliferate extensively, and differentiate into sperm cells or oocytes depending upon the embryo sex chromosomes composition [10].

Mammalian post-implantation development holds exponential embryonic growth by largely positive balance between cell proliferation against cellular demise. This intense proliferation accompanies irreversible events such as differentiation, migration or cell death [11]. The rapid and extensive embryonic morphogenesis from preimplantation development throughout gastrulation imposes faster cell turnover accompanied by drastic cellular phenotypic modulations [12,13]. Therefore, programmed cell death (e.g., apoptosis—see glossary) emerges as a physiological mechanism to sculpt the developing embryo—hijacking damaged or cell corpuses by engulfment—such as during cavitation processes [4,11,14,15,16,17]. Cell death may also be caused by non-cell autonomous factors [18,19]. The early embryo faces many life-threatening stressors—nutrient deprivation, pollutants, endocrine disruptors, self-generation of toxic metabolites, heat stress, etc.—that may be overwhelming for maintaining embryo viability [18]. Therefore, cells have multiple mechanisms to identify and counteract life-threatening conditions. For example, mammals have multiple layers of nutrient, organelle and nucleic acid sensing pathways for preserving cellular homeostasis [20,21,22,23].

**Figure 1:**
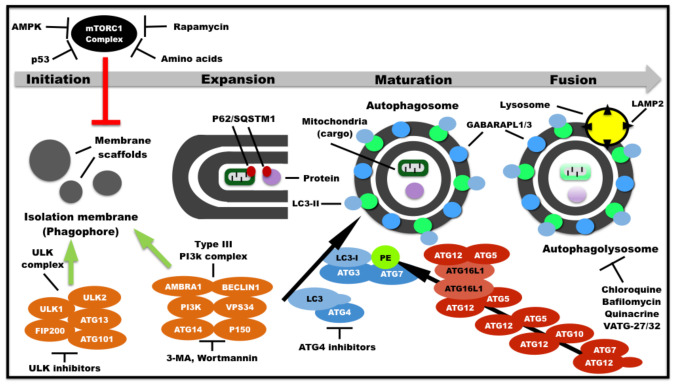
The mammalian autophagy pathway is a four-step process. Several environmental signals activate autophagy directly or indirectly, such as nutrient deprivation (e.g., amino acid depletion), DNA damage (p53-mediated transcription activation), developmental or environmental cues upstream of signaling pathways (e.g., adenosine monophosphate-activated protein kinase—*AMPK*), and mammalian target of rapamycin complex 1 (*mTORC1*) inhibitors (e.g., rapamycin). Autophagy initiation: cells exit homeostasis by inhibition the *mTORC1* complex and induction of autophagy activating kinase 1/2 *(ULK1/2)* complex (formed by *ULK1/2,* autophagy-related protein 13—*ATG13*, PTK2/FAK family interacting protein of 200 kDa—*FIP200*, and Autophagy-related protein 101—*ATG101* proteins) alongside type III phosphatidylinositol 3-kinase complex (formed by autophagy and beclin 1 regulator 1—*AMBRA1, BECLIN1, PI3K*, Vacuolar protein sorting 34—*VPS34*, beclin 1-associated autophagy-related key regulator/autophagy-related protein *14—ATG14,* mammalian homolog of yeast Vps15—*P150*). This activation forms an isolation membrane (phagophore) by sequestering part of a cellular membrane enclosing a fraction of the cytosol. The phagophore contains cargo such as organelles and/or macromolecules sequestered by adaptors such as sequestosome1 *(p62/SQSTM1)*. Expansion: the phagophore elongates and involves the cargo. The cysteine peptidase/autophagy-related protein *(ATG4)*, which is a component of the ubiquitin-like *ATG8* (Light-chain 3 *(LC3)*) system (blue), converts *LC3* into *LC3-I* by proteolytic cleavage at the C-terminus. Further, both autophagy-related protein 3 *(ATG3)* and autophagy-related protein 7 *(ATG7)* conjugate *LC3-I* with the lipid phosphatidylethanolamine (PE). The *LC3-I/PE* aggregate anchored at the autophagosome membrane becomes *LC3-II*. Similarly, gamma-aminobutyric acid receptor-associated proteins *(GABARAPLs)* are subject to proteolytic cleavage at the C-terminus by *ATG4* and conjugated to lipids by *ATG3/ATG7* for phagophore membrane binding*. LC3-II* and *GABARAPL* conjugates contribute to membrane elongation, cargo recognition, autophagosome edge closure, autophagosomal movement, and tethering to lysosomes. The ubiquitin-like autophagy-related protein 12 *(ATG12)* system also contributes to *ATG8* (*LC3* and *GABARAPL*) conjugation. It initiates with an *ATG12* cleavage by *ATG7* in an ATP-dependent manner. Furthermore, *ATG12* associates with autophagy-related protein 10 *(ATG10)*, thus forming another intermediate. Finally, *ATG12* conjugates with autophagy-related protein 5 *(ATG5)* and the latter interacts with autophagy related 16 like 1 *(ATG16L1).* Furthermore, a dimeric *ATG12-ATG5-ATG16L1* complex forms by *ATG16L1* homodimerization. This dimer complex contributes to *ATG8*-lipid conjugations. Maturation: The phagophore becomes a double-membrane enclosed vesicle named autophagosome. Fusion: the autophagosome fuses with a lysosome and becomes an autolysosome that allows complete degradation of cargo and release of cellular building blocks (e.g., amino acids and lipids) in the cytosol. The autolysosome formation requires the lysosome membrane-enriched glycoprotein lysosomal associated membrane protein 2 *(LAMP2)*. Stage-specific autophagy inhibitors were described. 3MA: 3-methyladenine.

Autophagy (see glossary) is an increasingly appreciated route that cells undergo to maintain viability while facing challenging conditions [24]. Autophagy or “self-eating” is an evolutionary conserved lysosome-mediated catabolic pathway for organelle and macromolecule recycling [25]. There are three types of autophagy: macroautophagy, microautophagy, and chaperone-mediated autophagy [24]. Macroautophagy (see glossary) remains by far the most elucidated mechanism, and from this point of the review thereafter, macroautophagy will be referred solely as autophagy. The execution of autophagy is a stepwise process orchestrated by several gene products coupled in multimeric protein complexes (Figure 1), which comprises four distinct stages [26,27]. Upon exit from cellular homeostasis (e.g., nutrient deprivation), the mammalian target of rapamycin complex 1 (*mTORC1*) dissociates from the *ULK1/2* complex releasing it to activate autophagy [28]. Free *ULK1/2* complex alongside the phosphatidylinositol 3-kinase (*PI3K*) complex form an isolation membrane known as phagophore. This occurs by sequestering part of a cellular membrane enclosing a fraction of the cytosol containing cargo such as organelles and/or macromolecules [29]. The phagophore elongates and becomes a double-membrane enclosed vesicle named autophagosome [26]. This autophagosome fuses with a lysosome to become an autolysosome that allows complete degradation of cargo and release of cellular building blocks such as amino acids and lipids in the cytosol [27].

There is an understanding that autophagy holds a basal activation at any given time [24]. This physiological autophagy activity can be interpreted as an evolutionary-driven strategy to maximize resources within the cell [24]. Upon exposure to stressors or environmental cues, cells modulate autophagic efflux to cope with fluctuations in nutrient availability, organelle numbers, macromolecule stocks, among other factors [25]. If cells do not recover from the initial stress-mediated damage, autophagy may continue to increase and become a life-threatening mechanism. The autophagy-mediated depletion of cellular constituents may lead to the so-called “autophagic cell death”, although such later definition remains challenged [30,31]. Hence, autophagy can also be selective leading to organelle-specific or macromolecule-specific catabolism [32,33,34].

Autophagy data are emerging at a fast pace in numerous experimental contexts and organisms [35,36,37]. Therefore, the aim of this review is to explore the contribution of autophagy to mammalian gametogenesis and preimplantation development while contemplating its contextual roles under environmental stressors and under assisted reproductive technologies (ARTs).

## 2. The Genetic Basis of the Autophagy Pathway

Most components of the autophagy pathway were first identified in yeast genetic screens. There are over forty annotated canonical autophagy-related genes (*Atg*) in *Saccharomyces cereviseae* and eighteen of them are required for autophagosome formation [38,39,40,41]. The mammalian *ATG* orthologs have been systematically interrogated in different cellular or developmental contexts [40,42], revealing essentiality or autophagy-dependencies. The use of reverse genetics has played a pivotal role in such undertaking, albeit almost exclusively in the mouse [40]. Knockout mice for autophagy genes (i.e., *Beclin1* and autophagy-related protein 5—*Atg5*) are viable and fertile [43,44]. This initial evidence suggested that gametogenesis and preimplantation development did not require autophagy. However, there is a caveat that emerges generating knockout mice by crossing heterozygous mutants: irrespectively of the genotype, embryos carry maternally derived protein throughout early development. These zygotic knockouts do not allow to unequivocally address the requirement of a candidate gene during early embryogenesis. Thus far, only two autophagy genes—*Atg5* and autophagy-related protein 7 *(Atg7)*—have been investigated using both oocyte and zygotic deletions [45,46]. These double oocyte/zygote mutants demonstrated that early embryogenesis requires autophagy. Further systematic investigation of other autophagy-related genes carrying both oocyte and zygotic deletions may contribute to dissect autophagy during early development.

The application of genome-wide tools accelerated the identification of autophagy-associated genes in mammalian cells. For instance, a proteomic approach searching for proteins interacting with 65 candidate autophagy genes found 409 associated proteins and 751 protein interactions under basal autophagy levels in human HEK293 cells, thus revealing an extensive autophagy regulatory network [47]. Upon inhibition of *mTORC1* complex with rapamycin, there was limited modulation of the autophagy regulatory network, potentially due to post-translational control or participation of unknown factors [47]. Alternatively, an unbiased genetic screening using short interfering RNAs for 21,121 human genes in an H4 neuroblastoma human cell line identified 236 genes as “hits”, which upon knockdown increased or decreased autophagy efflux using a *LC3-GFP* fluorescence readout [48]. Follow-up chemical screenings characterized the roles of these “hits” in the autophagy pathway and revealed several growth factors (e.g., *IGF-1, bFGF*) as inhibitors of autophagy activation by *type III PI3K* inhibition in an *mTORC1* complex-independent fashion [48]. Genome-scale studies continue to expand the autophagy regulatory network on multiple levels (transcriptomics, proteomics, metabolomics or applying multi-omics) under developmental and disease experimental settings [49].

Despite the growing number of autophagy-associated genes (non-canonical) in both yeast and mammals [50,51,52], the limited overlap among genome-wide studies suggests that the list is far from complete [52]. Unlike yeast that activates autophagy primarily under starvation conditions [53], mammalian cells modulate autophagy efflux under several biological processes and as response to an array of extracellular stimuli [50,51,54]. The understanding of how specific extrinsic signals (e.g., growth factor signaling) modulates the autophagy network will require substantial effort. One logical concern of comparing genome-wide studies is the bias from the species, cell type, and experimental factors. Indeed, there is evidence of species-specific and cell type-specific differences during autophagy activation at the regulatory network level. Under DNA damage, mouse embryonic fibroblasts activate autophagy under the p53-mediated apoptosis response [55]. The p53 transactivation activity is sufficient to induce several autophagy genes (*ULK1, ULK2, ATG4, ATG7*, and others) but the exact gene set and magnitude of gene expression modulation was affected by species (mouse embryonic fibroblasts vs. human fibroblasts) and cell type (fibroblast vs. HCT116 human colon cell line). Nonetheless, autophagy activation occurs in a *p53*-independent fashion [55]. This contextual interaction of *p53* and autophagy genes highlights the potential of network-based analysis to link autophagy to diverse biological processes, exemplified by the DNA damage response pathway. In turn, these genome-wide approaches remain challenging for germ cells and preimplantation embryos, although embryonic stem cells are an alternative model [15]. A comprehensive network analysis of autophagy-associated proteins in pluripotent cells should be highly informative.

## 3. Autophagy during Oogenesis

Gametogenesis is the process of formation of mature haploid gametes by cell divisions and stepwise differentiation during meiosis. It begins during fetal development in mammals, when PGCs migrate to the genital ridges before gonad formation [56]. PGCs proliferate extensively and differentiate into spermatogonia in males and oogonia in females [57,58]. Oogenesis involves the differentiation of PGCs into oogonias, follicle assembly, and growth, oocyte growth and maturation. The majority of follicle-enclosed oocytes (>99.9%) will never complete meiosis and most activated follicles undergo atresia while oocytes perish by apoptosis. Moreover, increasing evidence in mouse knockout models for core autophagy genes suggests that this process contributes to establishing and maintaining the oocyte pool in the ovary [59], as further described below.

The requirement for autophagy during oogenesis depends on the species (Table 1). In the mouse, autophagy activation occurs in the neonatal ovary [46], as found in other organs during the physiological starvation period after birth [44]. This autophagy efflux overlaps with a major loss in oocyte/follicle in neonatal female mice [46], albeit the underlying potential link remains unknown. Functional analysis showed that autophagy contributes to mouse oogenesis, since germ cell specific *Atg7* deletion resulted in ovarian insufficiency and subfertility [46]. The *Atg7* null females displayed diminished follicle counts after birth due to apoptosis. Curiously, the litter size of *Atg7* null females after the first gestation was initially indistinguishable from controls (~8 pups/litter), albeit later litters became smaller (~4 pup/litter) or females became infertile [46]. This complex phenotype of *Atg7* null females resembles the primary ovarian insufficiency in women aged <40 years and therefore could become an interesting animal model for this age-dependent human syndrome.

Further research demonstrated that autophagy contributes to oocyte maturation. Pig oocytes subject to in vitro maturation (IVM) accumulate and later degrade *LC3-II* protein [98], thus suggesting intensive autophagosome turnover. The knockdown of the pro-autophagy protein *BECLIN1*, in pig germinal vesicle oocytes compromised nuclear maturation, mitochondrial function, DNA integrity, and developmental competence [61]. In mice, *BECLIN1* protein accumulated throughout oocyte maturation but *Beclin1* mRNA knockdown disrupted oocyte cytokinesis, independently of autophagy [60]. Knockdown of the autophagy-related protein 14 *(ATG14)* gene did not affect oocyte nuclear maturation reinforcing that autophagy is not required for this stage of mouse oogenesis [60].

Pharmacological modulation also indicated the role of autophagy during oocyte maturation. Porcine cumulus–oocyte complexes (COCs) subject to IVM with the type III *PI3K* inhibitor 3-methyladenine (3MA) displayed lower cumulus cell expansion and oocyte arrest at the germinal vesicle stage [99]. Recently, maturation of bovine COCs with 3MA during IVM diminished oocyte competence as revealed by lower cleavage rate and development to the blastocyst stage [62]. Alternatively, the autophagy inducer rapamycin enhanced porcine oocyte maturation rates and embryonic development to the blastocyst stage. Rapamycin also increased blastocyst cell numbers (ICM, TE, and total cell count) and decreased apoptotic cell index [100]. Similar beneficial effects of autophagy induction were observed in pig morphologically poor oocytes under IVM with rapamycin [94].

The completion of oogenesis requires the interplay between oocytes and the follicle microenvironment [59]. Theca, granulosa, and cumulus cells are core cell types that make up ovarian follicles alongside oocytes contributing to the follicle microenvironment and oogenesis. Compelling evidence suggests that these cell types hold autophagy activity under contextual modulation. Human *BECLIN1* protein was detected in human theca cells in situ but not in granulosa and oocytes [101]. Nonetheless, *BECLIN1* protein also correlated with corpus luteum viability, thus marking theca-lutein and granulosa-lutein cells [101]. In turn, autophagy-related protein 8 (*ATG8*, also known as light-chain 3—*LC3*) was detected at the protein level in cumulus/granulosa cells of several species [62,98], irrespective of follicle size and developmental stage [102]. Exposure to FSH increased *LC3* protein accumulation in granulosa cells both in vitro and in vivo [102]. Under bovine oocyte IVM conditions, cumulus cells showed higher *LC3-I/LC3-II* conversion compared to oocytes [62]. It would be exciting to generate theca/cumulus/granulosa specific *Atg* deletions to determine the magnitude of oogenesis abrogation. For instance, granulosa-specific *Beclin1* ablation affected progesterone production in pregnant mice [103], albeit the phenotype was probably milder due to mosaicism in Cr-mediated recombination. In pigs, rapamycin induced higher *ATG5* and *LC3* levels in theca and granulosa [104].

Autophagy pathway also plays a role on oocyte survival after exposure to stress, such as post-ovulatory aging and heat stress. For porcine in vitro aged oocytes (an in vitro model of post-ovulatory oocyte aging), treatment with rapamycin for 24 h or 48 h of aging did not contribute to oocyte maturation (percentage of oocytes at metaphase II). However, treatment of 24 h-aged oocytes with rapamycin, increased the percentage of aligned chromosomes, normal spindles, and decreased ROS activity [105]. Consequently, 24 h-aged oocytes treated with rapamycin displayed cleavage and blastocyst rates similar to non-aged and non-treated controls. In addition, rapamycin also improved morphological blastocyst quality, total cell number, and decreased DNA fragmentation [105]. Autophagy was detected on growing oocytes of aged Japanese black cows (~12 years), which displayed oocytes of lower developmental competence. Nonetheless, the induction of mitophagy (see glossary) with the polyphenol antioxidant resveratrol improved oocyte quality and competence to blastocyst development in such aged cows [106]. However, during in vitro aging of mouse oocytes, up regulation of autophagy decreased some aging parameters, such as oxidative stress, spindle/chromosome abnormalities and cytoplasmic fragmentation, whereas autophagy down regulation aggravated these aging cellular phenotypes [63]. Exposure of bovine oocytes to heat shock during oocyte maturation has been shown to induce a series of detrimental effects reflecting on low oocyte developmental competence [64]. Heat shock induced *LC3* conversion in oocytes, while autophagy inhibition with 3MA enhanced the heat stress-mediated detrimental effects on oocyte developmental competence [62].

Oocyte cryopreservation is challenging due to the oocyte relative large size and abundant protein storage for sustaining early development. These traits complicate cell dehydration and increase the potential impact of protein damage diminishing oocyte viability and developmental competence. Cold stress during vitrification of mouse oocytes induced autophagy which was visualized by *LC3* staining [65,66]. Addition of 3MA to the vitrification process of immature oocytes decreased survival, nuclear maturation, and oocyte developmental competence [66]. In turn, vitrified metaphase II oocytes exposed to 3-MA during warming sustained developmental competence comparable to non-treated vitrified and fresh controls [65]. The use of conditional *Atg7*-deficiency in mice demonstrated that autophagy is not required for survival of mouse vitrified/warmed oocytes [67]. Autophagy induction with rapamycin before or after vitrification also did not necessarily improve cleavage and blastocyst rates [68]. These findings indicate a need for balancing degradation and anabolic processes after oocyte vitrification/warming, mature oocytes as more resistant to cryoinjury, or alternatively, rapamycin affected oocytes spindle migration [107] and lead to embryonic arrest.

## 4. Autophagy during Spermatogenesis

Autophagy contribution to spermatogenesis has also been explored (Table 1). Specific ablation of *Atg7* in mouse male germ cells led to subfertility and non-viable sperm with cytoskeleton damage and abnormal acrosome [69,70,71]. In turn, *Atg5* or *Atg7* knockout in mouse Sertoli cells led to low motility and non-viable sperm [72]. Depletion of *Atg5* or *Atg7* in rodent Leydig cells ultimately unbalanced testicular endocrinology, most notably in testosterone biosynthesis [69,73]. Autophagy deficiency in Leydig cells can affect male sexual behavior due to reduction in testosterone production [73]. Genetic and pharmacological ablation of *mTORC1* impaired cellular metabolism and protein secretion of epidydimal cells leading to mouse infertility [108]. These finding suggest that spermatogenesis requires autophagy in all cell types of the male reproductive tract.

Selective autophagy is emerging as an important process for sustaining sperm physiology. Lipophagy (see glossary) has been demonstrated during spermatogenesis for energy supply to male germ cells and supporting testosterone biosynthesis [69,73]. In addition, mitophagy has been detected in rat Sertoli cells [109] and reported to contribute to the clearance of sperm-borne mitochondria [110]. The physiological relevance of autophagy during spermatogenesis is not restricted to immature cells. Functional autophagy has been documented in mature sperm of several mammalian species by *LC3-I/II* conversion [78,79,80]. Although considered as a pro-survival mechanism for spermatozoa, the understanding of how autophagy protects sperm cells remains elusive.

Numerous stressors have been documented to affect spermatogenesis and testis function [69,79,111]. Processes such as energy restriction [74], heat stress [75], and pollutants [76,77] activated autophagy in sperm cells, Leydig cells, and Sertoli cells [72,76]. Autophagy has demonstrated contextual contributions to surmounting damage caused by endocrine disruptors to the male germ line, albeit the magnitude of protection depends upon the stressor [69]. Nonetheless, most stressors induced oxidative stress and acted throughout the mTOR downstream signaling [76,77,111]. There was also evidence that endoplasmic reticulum (ER) stress (see glossary) was coupled to autophagy activity during spermatogenesis [112]. More scrutiny on the roles of selective autophagy and uncoupling of stressors from their cellular effects (e.g., oxidative stress, DNA damage) should be informative.

In vitro culture of rat spermatocytes under different energy substrates (i.e., lactate, glucose or none) induced autophagy in contrast to their non-cultured counterparts [113]. This remains one of the few examples of how non-physiological in vitro conditions triggered autophagy. Cryopreservation is an ART that enhances autophagy in sperm [78,79]. Sperm storage at 5 °C suffices to activate autophagy [78], and thus could become a marker of more subtle sperm damage to improve semen cryopreservation protocols. Autophagy inhibition improved sperm viability after cryopreservation [80,81], thus suggesting that autophagy modulators per se may enhance the survival of cryopreserved sperm.

## 5. Autophagy during Fertilization

The sperm cell embarks on an intense remodeling event after gamete fusion in order to regain transcription and replication competence by active epigenetic reprogramming, removal of protamine, and acquisition of oocyte-derived nucleosomes [114]. Since multiple organelles inherited from sperm are cleared in the zygote, it may be possible that autophagy contributes to this extensive cytoplasmic turnover after fertilization (Table 1). Autophagy is much pronounced after fertilization in numerous species [115]. Evidence in *Caenorhabditis elegans* and mammals showed that sperm-borne mitochondria are prone to selective autophagy called mitophagy [82] or combined with the ubiquitin proteosome-mediated degradation pathway (see glossary) [83,84,85,86], although some reports do not support such findings [87]. Further research should resolve the extent of autophagy versus ubiquitin proteasome-mediated degradation during fertilization in mammalian species [88]. Despite the underlying recycling mechanisms, sperm remodeling occurs at a fast pace and paternal mitochondria fail to contribute to the developing embryo. Whether post-fertilization calcium oscillation triggers autophagy persists as an interesting question yet to be addressed [115]. Finally, it remains not fully explored whether ARTs (e.g., in vitro fertilization or intracytoplasmic sperm injection—ICSI) or stressors affect organelle turnover after fertilization. An initial report found retained mitochondrial sheath in mouse morulae obtained from ICSI [87].

## 6. Autophagy during Preimplantation Development

**Figure 2:**
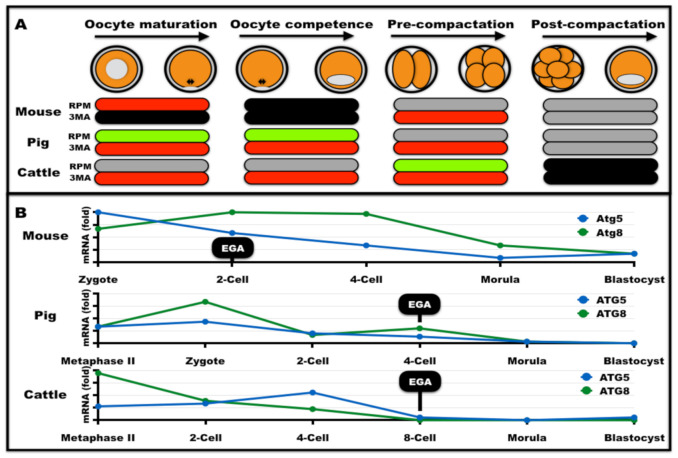
Autophagy modulation on mammalian oogenesis and preimplantation embryonic development. (**A**) In vitro incubation of oocytes/embryos with rapamycin (RPM; *mTOR* inhibitor/autophagy inducer) or 3-methyladenine (3MA; *Type III PI3K* inhibitor/autophagy inhibitor). Green bar: stage-specific improvement in oocyte maturation (progression from germinal vesicle to metaphase II stage), oocyte competence (ability of an oocyte to give rise to a blastocyst), and preimplantation embryonic developmental potential at the pre-compactation (zygote to 4-cell) and post-compactation (beyond 4-cell) stages (increase in cleavage and blastocyst yields, respectively). Red bar: stage-specific decrease in oocyte maturation, oocyte competence, or embryonic developmental potential. Gray Bar: no observable effect. Black bar: not determined. (**B**) Schematic representation of mRNA levels for the autophagy genes autophagy-related protein 5 *(ATG5)* and autophagy-related protein 8 *(ATG8/LC3)* during preimplantation embryonic development in selected mammalian species. Black boxes highlight the embryonic genome activation (EGA) for each species (the EGA in cattle occurs in the eight-to-sixteen cell stage transition).

The roles of autophagy during preimplantation development remain under intense investigation (Table 1). The impaired post-fertilization development of germ cell and zygotic *Atg5*-null mouse embryos between the four-cell and eight-cell stages showed that these stages require autophagy [45]. Remarkably, the overall protein synthesis was reduced by 30% in *Atg5*-null embryos [115]. This was also supported by the fact that the four-cell and eight-cell stages overlap with a major burst of protein synthesis in the mouse embryo [86]. Despite this temporal connection between protein turnover and autophagy dependency, the casual link between the two phenomena requires further investigation. Partial rescue from embryonic arrest was attained by fertilization of *Atg5*-null eggs with wild-type sperm cells [45]. The limited rescue of embryonic development in these heterozygous embryos was likely due to delayed autophagy activation, since *Atg5* protein became available in these embryos from the two-cell stage onward. After this seminal report, much progress has been made in deciphering autophagy in early embryos (Figure 2). Lysosome depletion by injection of short interfering RNAs into mouse zygotes caused embryonic arrest at the two-cell stage, while chemical inhibitors caused arrest at the four-cell and eight-cell stages or at the morula stage [116]. This later report suggested that lysosome depletion partially phenocopies the developmental block caused by autophagy inhibition. Mechanistically, autophagy activation after fertilization is independent of *mTOR* signaling but relies on yet unidentified factors associated with the *PI3K* pathway [86,117].

During preimplantation embryonic development, the mRNA abundance of mouse, pig, and cattle autophagy genes (e.g., *Atg5* and *Atg8/Lc3*) sharply and steadily decreases from the zygote to the blastocyst stage (Figure 2; [89,90,91]). Qu et al. [15] used an embryonic stem cell-based model of embryonic development (i.e., cystic embryoid bodies that resembled blastocysts) to show that autophagy is required for embryo cavitation. Both *Atg5* and *Beclin1* knockout failed to cavitate due lack of “cell corpuses” clearance, despite intact programmed cell death machinery. Further evidence came from embryo-driven work indicating that non-physiological autophagy levels impaired ICM and TE segregation [89,92].

Autophagy dependency has been extensively carried out in mammalian early embryos by pharmacological means. As expected, blockage of autophagy by 3MA from zygote-stage onward or from zygote to the two-cell stage, respectively, did not fully arrest embryogenesis (mouse, pig, and cattle) albeit blastocyst yields dropped substantially [89,90,91]. Of note, treating embryos with autophagy modulators (i.e., 3MA and rapamycin) after their autophagy-dependency (e.g., two-cell, four-cell) did not affect developmental potential or embryo cell numbers [89,91]. This finding suggests limited toxicity and post-compactation development in an autophagy-independent manner (Figure 2). Moreover, autophagy modulators lead to fluctuations in gene expression levels of both ICM-specific and TE-specific transcription factors [89,91]. For instance, increasing autophagy by rapamycin leads to TE-specific autophagy and beclin 1 regulator 1 *(AMBRA1)* activation and *LC3* in a rather stochastic fashion [92]. Both 3MA and rapamycin decreased mitochondrial DNA copy numbers and enriched for mitochondria altered morphology and increased autolysosome numbers in mouse embryos [89]. Dose-dependent effects of rapamycin were found to beneficial for blastocyst yield, inhibition of apoptosis and ICM/TE cell numbers [90], thus reinforcing the need for detailing the selection of autophagy modulator concentrations. In cattle, enhanced autophagy by rapamycin supplementation compensates, at least partially, for ER stress [90]. Although Song et al. [90] raised the link between in vitro embryo production and autophagy, it remains to be described the extent of autophagy induction by such non-physiological conditions in comparison to the in vivo environment.

A link between mitochondrial stress and autophagy was made in the pig [118,119]. Exposure of pig embryos to high hydrogen peroxide (H_2_O_2_) concentrations induced microtubule associated protein 1 light chain 3 beta (*MAP1LC3B*) and lysosomal associated membrane protein-2 (*LAMP2*) gene expression and *LC3* accumulation [118]. The phosphatase and tensin homolog-induced kinase 1 (*PINK1*)—*Parkin* coupled mechanism is also required for mitochondrial homeostasis in pig embryos, since *PINK1* depletion by mRNA knockdown increased abnormal mitochondrial content and induced autophagy and mitophagy [119]. It has been revealed that poly(ADP-ribosylation (*PARP*) is tied to selective autophagy of ubiquitinated proteins in pig blastocysts [120]. *PARP-1* inhibited *mTORC1* by decreasing p-p70S6K-thr389 phosphorylation and sustaining *ATG5, BECLIN1*, and *LC3* gene expression [120]. Inhibition of *Sirtuin*-class histone deacetylases (HDACs) also lowered pig blastocyst development and cell numbers while accompanied by autophagy activation [121]. This *Sirtuin* deficiency induced *LC3* and may have affected the acetylation status of other *Atg* genes and *FoxO*-class transcription factors [121,122]. Another example of selective autophagy during preimplantation development is lipophagy [123]. An engineered approach using a *p62*-LD domain fusion protein partially depleted mice embryos from lipid droplets revealing that such structures are necessary for embryogenesis and for fine-tuning overall lipid content [86,123]. A rather curious fact was the observation that removal of amino acids from culture medium did not affect lipid droplet depleted embryos in contrast to wildtype controls [123]. Since lipid droplet content varies between species, it would be interesting to run similar experiments with embryos of several livestock species.

Preimplantation embryos are highly susceptible to metabolic perturbations [92]. Mouse embryos displayed basal autophagy that was enhanced by glucose [92,124]. Exposure of mouse embryos to diabetes-like milieu (i.e., 20–52 mM glucose) led to increased embryonic arrest, higher apoptotic index, fetal resorptions, and malformations [92,93]. The hyperglycemic-mediated autophagy induction accompanied by enhanced *GAPDH* activity lowered intracellular glucose content in exposed blastocysts but could not compensate for most of the damage [92]. It would be interesting to address if this heterogeneous autophagic response in mouse blastocysts is based on cell intrinsic factors, such as differential metabolism or programmed cell death responsiveness [14], altered expression of lineage-specifying genes [89] or overlaps with single-cell glucose content. Alternatively, exposure of sheep blastocysts to the environmental contaminant polychlorinated biphenyls reduced cell number, increased the proportion of TUNEL-positive blastomeres positive (marker of DNA damage) but failed to elicit the expression of all autophagy genes tested [125]. However, it is early to describe as a “failed autophagy activation scenario” since no post-transcriptional analysis was carried out or determined in a side-by-side comparison to another autophagy-inducing condition. In turn, delayed implantation in ovariectomized mice showed autophagy activation in blastocysts due to estradiol and progesterone deprivation [89,126]. Of more broad importance, it has been postulated that autophagy induction during delayed implantation may have long-lasting detrimental consequences on fetal viability [79,89]. Therefore, it remains to be explored if modulation of autophagy activity may contribute to enhancing implantation rates, offspring delivery or correlates with such parameters under the context of environmental stressors or ARTs.

There is accumulating evidence that ARTs leads to autophagy modulation in early embryos. A single report from an unbiased gene expression analysis has found that gamma-aminobutyric acid receptor-associated proteins (*GABARAPLs*) gene transcripts are negatively enriched in human ICSI-derived blastocysts under the influence of the paternal age [127]. The availability of genome-wide gene expression profiling data from human embryos [128] could be used to charting the expression of autophagy-related genes throughout human early embryogenesis. Embryo cryopreservation is another potential area to explore autophagy, due to its application in livestock breeding programs and human assisted reproduction. Autophagy was detected in blastocysts derived from vitrified zygotes [129], although its induction level did correlate with full-term developmental potential of cryopreserved embryos. Further work should elucidate the roles of autophagy in the survival of cryopreserved preimplantation embryos.

Somatic cell nuclear transfer (SCNT) allows cellular reprogramming of a differentiated nucleus by an enucleated oocyte [130]. However, cloning remains labor-intensive [131] and holds a relatively low efficiency caused by both technical and biological factors [130,132]. Moreover, fertilization-mediated autophagy induction was not observed in SCNT mouse embryos following chemical oocyte activation [95]. This observation seems contextual to SCNT embryos since mouse activated oocytes showed autophagy induction [45]. This post-activation inhibition of autophagy was due to actin filament depolymerization by cytochalasin incubation during chemical activation of SCNT reconstructed oocytes, while rapamycin restored autophagy and led to the recovery of cloned preimplantation development [95]. The enhanced autophagy in SCNT embryos had pleiotropic effects: stimulated maternal mRNA degradation, accelerated active DNA demethylation in pseudo-pro-nuclei, and increased blastocyst yields [95,133]. It remains to be investigated if such improvements in SCNT embryos impact full-term development. In contrast, rapamycin supplementation during cytochalasin-containing pig oocyte activation did not exert a similar beneficial effect on blastocyst development [134]. It remains unknown if the benefits of autophagy in SCNT embryonic development overlap with the benefits of HDAC inhibition or act in different ways.

Pig SCNT showed different autophagy activation kinetics in comparison to control parthenotes [94,96]. Autophagy induction was more pronounced at the pseudo-pronuclear and the two-cell stage in SCNT pig embryos compared to the controls. Autophagy intensity was inverted at the four-cell stage but similar in blastocysts, pointing out to faster autophagy kinetics in clones [94]. A side-by-side comparison of these two groups with IVF-derived embryos would be informative, due to more physiological autophagy induction with sperm-induced oocyte activation. The *LC3* knockdown with short interfering RNAs in pig SCNT embryos showed that autophagy selectively modulates mRNA abundance—and most likely the protein content—of components of the DNA methylation machinery (*DNMT1*, *DNMT3B*), pluripotency-associated transcription factors (TFs; *DPPA3/STELLA* and *STAT3*), kinases (*c-MOS*), *TGF-β* pathway (*BMP15* and *GDF9*) but not the core pluripotency TFs *OCT4* and *SOX2* [96]. Rapamycin was able to augment the relative abundance of several embryonic genes and autophagy-associated genes in SCNT embryos [94,96]. In turn, rapamycin supplementation during pig oocyte IVM improves SCNT and parthenote blastocyst yields (but no effect on total cell numbers), albeit from low-grade oocytes only, thus strongly suggesting that it strictly recovers oocyte competence [94]. Vitamin C or rapamycin increased autophagy in bovine SCNT embryonic development up to the blastocyst stage [97]. The incubation of bovine SCNT embryos after activation with the histone 3 Lysine 9 trimethylation (H3K9me3)-specific methyltransferase inhibitor chaetocin increased embryonic development (cleavage and blastocyst rates) and embryo quality (cell numbers and apoptosis index) concomitant with increased autophagy gene activity at mRNA and protein levels [133]. These results reinforce a connection between autophagy and epigenetic reprogramming. A single-cell genome-wide transcriptome analysis of two-cell and four-cell mouse SCNT embryos also revealed autophagy as a roadblock to cellular reprograming [135]. These facts support that autophagy plays an important role during embryonic development after SCNT. Investigations that combine both functional and cellular–molecular assays should elucidate the missing links between developmental competence, chemical activation effects, and cellular reprogramming-intrinsic factors to overall SCNT embryo development.

## 7. Future Directions

The integration of the autophagy data from gametogenesis and preimplantation development gave a broader picture of its contextual roles during development. It becomes clear that the importance of autophagy is dictated by developmental states as well as interactions with stressors and ARTs. More studies with side-by-side comparisons between physiological and stress/ARTs conditions will determine the magnitude of autophagy induction and its potential contribution to sustain cellular viability and function.

**Figure 3:**
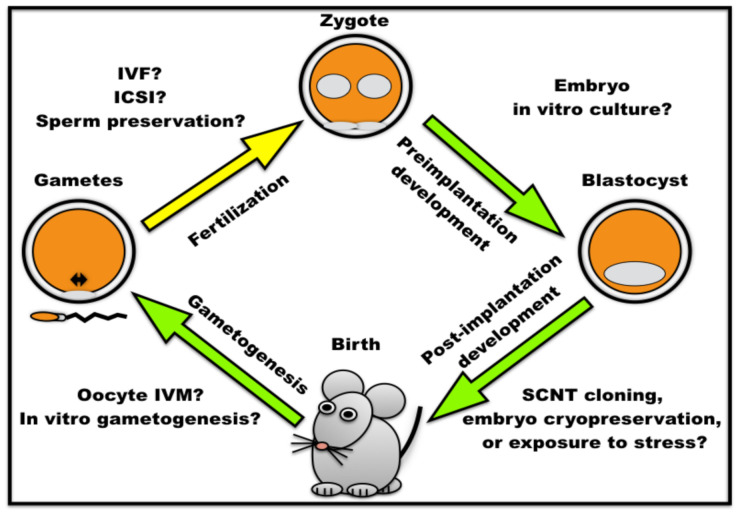
Potential contextual roles of autophagy during mammalian development. Autophagy is required during preimplantation embryonic development (zygote to blastocyst; green arrow) but the impact of in vitro embryo culture remains unknown. Post-implantation development requires autophagy (blastocyst to birth; green arrow) albeit it remains unclear if more challenging conditions to full-term development (e.g., cloning, embryo cryopreservation or exposure to in vivo stress) require autophagy. Autophagy is required for in vivo gametogenesis (animal to gametes; green arrow), although the impact of oocyte in vitro maturation (IVM) and in vitro gametogenesis would be informative. Autophagy during fertilization (gametes to zygote; yellow arrow) and sperm remodeling remains controversial. An attractive opportunity to contribute to this discussion is to determine if in vitro fertilization (IVF), intra-cytoplasmic sperm injection (ICSI), and sperm preservation methods (cooling, freezing or lyophilization) affect fertilization and the associated processes. SCNT: somatic cell nuclear transfer.

The literature review described here points out unaddressed questions for future research (Figure 3). The dose-dependent effects of autophagy modulators reinforce the potential for planning further investigations with multiple concentrations or incubation time. Mechanistic detailing by application of multiple inhibitors at divergent points of the autophagy cascade could be more informative, such as to distinguish between overall organelle stocks and the incidence of damaged ones. Furthermore, autophagy monitoring has relied mostly on *LC3* reporter assays, which act as an autophagosome marker [40]. This strategy requires lysosome inhibitors to distinguish between autophagosome accumulations during autophagy efflux from inhibition of autophagic degradation [40,136]. Several studies lack this control, thus reinforcing the need for adequate experimental design and application of alternative autophagy reporter assays. Another exciting research possibility is to explore intrinsic cellular variability for autophagy activity, as found for preimplantation embryo viability [137]. Single-cell analyses could also reveal potential autophagy variation under physiological conditions or stress/ARTs. This intrinsic variation may be driven by yet undefined factor(s) or be stochastic in nature, which would be a finding of paramount importance. As for many other biological questions, single-cell analyses could also resolve conflicting data, as exemplified by the unclear roles of autophagy induction in mature sperm. Finally, the exploration of autophagy for longer periods should increase the understanding of its long-term effects on cellular physiology.

## 8. Concluding Remarks

The endeavor of understanding autophagy during mammalian gametogenesis, fertilization, and preimplantation development has rapidly being fulfilled by accumulating information in numerous cell types and organisms. The downside is that progress has been made almost exclusively for macrophagy using low resolution tools. Further mechanistic insight and network-based approaches are thus needed to promote a shift from a relatively fixed dichotomy of “all or none autophagy” phenomenon to more contextual autophagy investigations. Novel experimental designs (e.g., encompassing combined environmental stresses with ARTs or multiple autophagy modulators) and novel experimental tools should also contribute to a more detailed understanding of contextual autophagy in mammalian preimplantation embryos and the germline.

## Figures and Tables

**Table 1:** Context-dependent roles of autophagy from gametogenesis to preimplantation embryonic development.

Event	Physiological Role	Stress	ARTs
Oogenesis	Required for mouse oogenesis (except for oocyte maturation) [46,60] and for bovine and pig oocyte maturation [61,62]	Activated during oocyte aging [63] and environmental stress (e.g., heat stress) [62,64]	Contributes to oocyte survival after vitrification [65,66,67] but rapamycin was detrimental to developmental competence after warming [68]
Spermatogenesis	Required for testosterone production, sperm and acrosome formation, motility, and fertility [69,70,71,72,73]	Activated during environmental stress [74,75,76,77]	Activated by sperm cooling and freezing [78,79]. Autophagy inhibition improved sperm viability [80,81]
Fertilization	Ongoing debate if it recycles sperm-borne mitochondria [82,83,84,85,86,87,88]	-	-
Preimplantation embryonic development	Required for post-compactation embryogenesis in the mouse [45,89], cattle [90], and pigs [91]	Activated under environmental stress [92,93]	Decreased or delayed activation in SCNT embryos [94,95,96]. Autophagy inducers increased SCNT reprogramming to blastocysts in mice [95], pigs [94,96], and cattle [97]

ARTs: assisted reproductive technologies. SCNT: somatic cell nuclear transfer.

## Data Availability

This review did not require generating or analyzing datasets.

## Glossary

Apoptosis: programmed cell death pathway characterized by cellular changes such as cell shrinkage, genomic DNA fragmentation, and cytoplasmic fragmentation.

Autophagy: pro-survival lysosome-mediated cytoplasm degradation pathway for organelle and macromolecule recycling.

Endoplasmic reticulum (ER) stress: accumulation of misfolded proteins in the ER that leads to activation of the unfolded protein response to reduce protein synthesis and improve protein folding and stabilization.

Lipophagy: selective autophagy targeting lipid droplets.

Macroautophagy: autophagy process in which cellular contents are engulfed by membrane-derived structures and degraded by lysosomes for recycling.

Mitophagy: selective autophagy targeting mitochondria.

Ubiquitin-mediated degradation: process of protein ubiquitination that leads to degradation by the 26S proteasome pathway.

3MA	3-methyladenine
ARTs	assisted reproductive technologies
ATG	autophagy-related genes
EGA	embryonic genome activation
ER	endoplasmic reticulum
ICM	inner cell mass
ICSI	intracytoplasmic sperm injection
IVM	in vitro maturation
SCNT	somatic cell nuclear transfer
TE	Trophectoderm
TFs	transcription factors
